# The Burden of Motorcycle Crash Injuries on the Public Health System in Kisumu City, Kenya

**DOI:** 10.9745/GHSP-D-22-00197

**Published:** 2023-02-28

**Authors:** Wilberforce Cholo, Wilson Odero, Japheths Ogendi

**Affiliations:** a Department of Public Health, Maseno University, Kisumu, Kenya and Department of Public Health, Masinde Muliro University of Science and Technology, Kakamega, Kenya.; b School of Medicine, Maseno University, Kisumu City, Kenya.

## Abstract

**Background:**

In Kenya, the increased use of motorcycles for transport has led to increased morbidity, mortality, and disability. These injuries exert a burden on the public health system, yet little information exists on health care resource usage by motorcycle crash injury patients. We aimed to estimate the burden of motorcycle crash injuries on the health system in Kisumu City.

**Methods:**

We conducted a 6-month prospective study of all motorcycle crash injury patients who presented to 3 Tier III public and private hospitals in Kisumu City between May and November 2019. We collected data on demographics, emergency department (ED) visits, admissions, anatomic injury site, services used, and injury severity. We reviewed hospital records to obtain denominator data on all the conditions presenting to the EDs.

**Results:**

A total of 1,073 motorcycle crash injury cases accounted for 2.0%, 12.0%, and 13.6% of total emergency visits, total injuries, and total admissions to the hospitals, respectively. Men were overrepresented *(P*<.001). The mean age was 29.6 years (±standard deviation [SD] 12.19; range=2–84). The average injury severity score was 12.83. Surgical interventions were required by 89.3% of patients admitted. Of the 123 patients admitted to the intensive care unit, 42.3% were due to motorcycle accident injuries.

**Conclusion:**

Motorcycle injuries impose a major burden on the Kisumu City public health system. Increased promotion and reinforcement of appropriate interventions and legislation can help prevent accidents and mitigate their consequences. Focusing on motorcycle injury prevention will reduce accident-related morbidity, hospitalization, severity, and fatalities and the impact on the public health system.

## Abstract (precis)

Reliable estimates of motorcycle-related injuries and hospitalization, as well as their impact on the health system, are needed for evidence-based policymaking, advocacy, and priority-setting for appropriate and effective interventions, resource mobilization, and future research.

## Teaser

**Key Findings:**

- Motorcycle crash injuries were the second most common cause of both emergency department visits and hospital admissions.
- The findings showed that motorcycle injury cases contributed to greater radiological diagnostic service use, operating theater use for minor and major surgical procedures, and intensive care unit admissions, demonstrating the extent to which motorcycle crashes contribute to increased use of public health system resources.

**Key Implications:**

- Documentation of motorcycle-related injuries and hospitalizations and their impact on health services is essential for evidence-based policymaking, advocacy, and priority-setting for interventions, resource mobilization, and future research.
- Policymakers, hospital administrators, and trauma center managers should consider motorcycle injuries when planning for emergency trauma service delivery, including human resources, bed capacity, equipment and supplies, and operations.
- Lack of helmet use by more than 80% of motorcycle riders and passengers and their increased usage of hospital resources convey an urgent need for improved preventive strategies and enforcement.

## INTRODUCTION

Motorcycle injuries are a public health problem globally.^1^ The effects of motorcycle injuries in low- and middle-income countries (LMICs) are twice as high as those in high-income countries.^2^ These injuries contribute significantly to mortality and morbidity, placing a significant economic burden on individuals injured, the public health system, and governments, as shown by lost wages, unemployment, long-term medical expenses, and intangible suffering.^2^ Globally, motorcycle fatalities account for 28% of all road traffic deaths.^1^ In the World Health Organization Africa region, between 7% and 16% of all road traffic deaths are motorcycle-related deaths.^1^

During a crash, motorcycle riders and their passengers endure a high-energy impact caused by the collision. Absorbing this energy causes injuries to the head and brain, extremities, and spine, making crash victims more susceptible to severe injuries and fatalities, in addition to longer hospital stays, more intensive care unit (ICU) admissions, and high expenses for care.^3^^,^^4^ Motorcycle injuries strain a country’s health system and health finances, necessitating the use of additional resources and increased levels of care.^5^ Previous studies from Brazil, Jamaica, Rwanda, Taiwan, and the United States reported that 65%, 73.6%, 50%, 31.6%, and 36% of motorcycle injury cases, respectively, were admitted to the hospital, of which 95%, 71.6%, 71.6%, 31.6%, and 36% required surgical interventions, respectively.^6^^–^^10^ Furthermore, public health systems are not adequately equipped to meet the additional trauma care needs and face major gaps in coordinated emergency responses.^11^ Traditionally, public health resources have been prioritized for health system development for communicable diseases.^12^

In Kenya, the use of motorcycles for public transport has increased by more than 33% in just 15 years, from 6% in 2005 to 39.6% in 2020.^13^^,^^14^ Many people prefer motorcycles as a mode of transport because they offer a fast means to get around in cities, towns, and rural areas; are efficient in navigating around traffic jam delays; and are available day and night.^15^ The rise in motorcycle crash injuries has been attributed to excessive speeding, overloading for quick financial returns, riding under the influence of alcohol, the poor quality of roads, and improper passing of other vehicles. In addition, most motorcycle riders disregard traffic rules, lack the appropriate riding competencies, and seldom wear helmets.^15^^,^^16^ Motorcycle crash injuries exert additional pressure on the Kenyan health system, which is poorly equipped to provide the needed health care services.^17^ Hospital morbidity statistics in Kenya show that injuries from motorcycle crashes account for 2%–3% of all hospital visits, 22%–64% of trauma admissions, and 50%–52% of surgical interventions.^18^^,^^19^ In Kisumu City, where the incidence of motorcycle crashes ranges from 41% to 62%, there are limited data on the burden of motorcycle injuries on the public health system in the city.^20^^,^^21^

The incidence of motorcycle crashes ranges from 41% to 62% in Kisumu, but there are limited data on the burden of motorcycle injuries on the public health system in the city.

Motorcycle helmets are known to reduce the risk of death and head injury among motorcycle users in crashes,^22^ but their use in Kenya has been dismal.^23^^,^^24^ Consequently, in 2012, advocacy by local nongovernmental organizations and civil organizations led legislators in Kenya to amend the traffic bill that reinforced the mandate of helmet use and other safety measures among all motorcyclists and their passengers.^25^^,^^26^ Despite this enactment, helmet use has not increased substantially.^27^ The Kisumu county government has also integrated road safety intervention strategies, including mandatory helmet use by motorcycle users, into its development plan.^28^

Although these safety measures are pivotal in reducing injuries and deaths, prehospital care can also help to minimize the disabilities, sequelae, and lethality of crashes.^29^ Studies have shown that quality prehospital care and hospital care prevent and reduce the probability of death and sequelae.^30^^,^^31^ However, Kenya has an inadequate prehospital care system to provide emergency transportation and injury management.

Reliable estimates of motorcycle-related morbidity, hospitalization, severity, and fatalities, as well as their impact on the public health system, are essential for evidence-based policymaking, advocacy, and priority-setting for appropriate and effective interventions, resource mobilization, and future research.^32^^–^^34^ Unfortunately, health information systems are inadequate in many cities, including Kisumu, in LMICs.^21^ No published information exists on the burden imposed on health services by different types of motorcycle crash injuries and severity levels in Kisumu City. The few studies on motorcycle injuries in the region have been population based.^20^^,^^21^ Moreover, no studies have been carried out in more than 1 hospital. Understanding the magnitude of motorcycle crash injuries and their patterns is essential for implementing prevention strategies and treatment protocols.^4^

This study sought to provide data on motorcycle crash injuries requiring emergency department (ED) visits and/or hospitalization and estimate the burden on Tier III hospitals in Kisumu City, Kenya.

## MATERIALS AND METHODS

### Study Site

This study was conducted in Tier III hospitals in Kisumu, a city in western Kenya. Kisumu is the third largest city in Kenya after Nairobi and Mombasa and has a population of 610,082 persons with an annual growth rate of 2.8%.^35^ More than half (52%) of the population are aged 0–19 years, and 36.2% are aged 15–45 years.^35^

Health services in Kisumu are available through a multifaceted system consisting of the central government, local authorities, nongovernmental organizations, church missions, and an array of private medical practitioners and traditional healers.^36^ Altogether, Kisumu has a total of 15 public and private health facilities.^35^ This study focused on 3 Tier III hospitals: Jaramogi Oginga Odinga Teaching and Referral Hospital, Kisumu County Hospital, and Aga Khan Hospital. The first 2 are public hospitals run by the Kisumu county government,^29^ and Aga Khan Hospital is privately run. We selected these hospitals because they have active EDs and operating theaters that provide care to injury patients and operate on a 24-hour basis. The top 5 leading causes of hospitalization are malaria (16.7%), skin diseases (13.5%), respiratory diseases (11.2%), diarrheal diseases (7.3%), and road traffic injuries (5.9%).^35^^,^^36^ The main mode of transportation used within Kisumu City is walking, comprising 53% of daily trips.^37^ Motorcycles, matatus, and bicycles comprise 19%, 13%, and 4%, respectively, of urban transport.^37^ Motorcycle-related deaths and injuries are a major public health problem in Kisumu City.^15^

### Study Design and Population

We conducted a 6-month prospective descriptive study of all motorcycle injury patients who presented to 3 Tier III public and private health facilities in Kisumu City from May 6, 2019 to November 6, 2019. We also reviewed medical records to obtain data on all emergency presentations and injury cases within the same period.

### Sample Size Determination

A priori power analysis was used to determine the sample size (n),^38^ computed as a function of the required power level (1-β) which was taken as 95%, the pre-specified significance level (α=0.05), and a population effect size of 10%. An a priori analysis provides an efficient method of controlling statistical power before a study is conducted.^32^ Based on this analysis, the sample size was 1,073 participants (Table 1).

**TABLE 1.** Sample Size of Motorcycle Crash Injury Cases in Kisumu, Kenya, by Hospital

**Hospital**	**Cases per Day, No.** **(n=6)**	**Cases Over 6 Months, No.** **(n=1,073)**
Jaramogi Oginga Odinga Teaching and Referral Hospital	3	613
Kisumu County Hospital	2	310
Aga Khan Hospital	1	150

### Data Collection

Data were obtained on all the motorcycle crash injury cases that presented to the accident and emergency units of the hospitals over the study period. The study was conducted from registration desks and examination rooms in EDs of the participating hospitals. We recruited successive motorcycle trauma patients who presented to the hospitals from May 6 to November 6, 2019. On arrival, all motorcycle injury patients were informed of the study by the registration clerk after being examined by either a doctor or a clinical officer. Injured patients gave their informed consent either after they were stabilized or relatives who brought motorcycle injury cases for care assented. Patients were initially assessed according to Advanced Trauma Life Support guidelines and contemporary standards of trauma care. They were interviewed by a survey assistant who completed a structured questionnaire eliciting personal and injury circumstances. We obtained information on sociodemographic characteristics, category of road user (rider, pillion passenger, or pedestrian), mechanism of injury, day of the week injury occurred, time of the injury, helmet use, diagnostic procedures performed, injury disposition, and treatment procedures conducted. For patients who were admitted, we recorded hospital bed days, use of ICU, type of treatment given, and injury outcome from the inpatient notes. Casualties who were admitted and discharged on the same day were considered to have been hospitalized for 1 day. Data on anatomic site of injury and diagnosis were also collected: the pelvic girdle and the rest of the lower limb were categorized as lower extremities; the pectoral girdle constituted upper extremities; head injuries constituted injuries to the face, neck, and head (including concussions); and thoracic injuries including rib fractures and visceral organs within the thorax were considered as chest injuries.

For each casualty, the hospital attending physician assessed the severity of injury sustained using the abbreviated injury scale (AIS) and injury severity score (ISS) scale based on the clinical diagnosis. The ISS measures the severity of the injury based on the AIS, which was quantified by taking the highest score in the 3 body regions most affected. The ISS score ranges from 0 to 75^39^^,^^40^ and was categorized as follows: ISS 1–8, 9–15, 16–24, and 24+. We classified injuries using the following injury groups: pelvic injury, hip fracture, and tibia fracture/complex foot fracture or distal/shaft femur fracture (lower extremity injury groups); shoulder and upper arm injury, and radius, ulna, or hand fracture (upper extremity injury groups); mild traumatic brain injury (TBI) (AIS 1–2), serious TBI (AIS 3), and severe TBI (AIS≥4) (head injury groups); face injury group; thorax injury and rib fracture (thorax injury groups); mild abdominal injury (AIS≤2) and severe abdominal injury (AIS≥3) (abdomen injury groups); and spinal cord injury and stable vertebral fracture/disc injury (spine injury group). We further divided them into minor and major injuries. Therefore, motorcycle injuries were classified into minor (ISS<16) and major injuries (ISS≥16).^41^

Patients were followed from the time of presentation to discharge from the hospital, referral, or death. We documented the management of patients, final outcome, and available postmortem findings for mortality cases. We also accessed and reviewed records for all emergency and injury cases that presented within the same period to enable assessment of the burden of motorcycle injuries on selected health services in the hospital. Data collected were total number of emergency cases, emergency admissions, radiological investigations, and surgical procedures. We compared these with motorcycle injury cases to establish the extent or magnitude of usage compared to other emergency cases, including assaults, other road traffic cases, falls, and other injuries consisting of drownings, pricks, bites (e.g., dog or snake), and burns. We obtained data on medical costs from bills served to the patients from the hospital billing section. We sought and obtained information on the following costs and fees: first aid services, consultation or observation, laboratory tests, radiological tests, medicines, surgical procedures, hospital bed per day, and food. These were summed up for each injury patient as the cost of treatment for the period of stay in the hospital accordingly. We also included the costs of transport to the hospital as reported by the patient or caretakers and ambulance where applicable.

### Data Analysis

We coded the quantitative data and entered it into SPSS version 21. We used descriptive statistics to examine usage of services by motorcycle injury cases, anatomic injury site, and severity in relation to road user. We used Chi square to identify factors associated with radiological services. Odds ratio and its confidence were estimated using a generalized linear model to assess factors contributing to the burden on hospital services. For all tests, the threshold of significance was *P*<.05.

### Ethical Approval

Ethical and research approvals were obtained from Maseno University Ethics Review Committee and Jaramogi Oginga Odinga Teaching and Referral Hospital Ethical Review Committee. We received permission from each of the participating hospitals to collect data. All participants gave their informed consent before enrollment.

## RESULTS

### Proportion of Motorcycle Crash Injuries in Relation to Other Injuries in ED Visits

A total of 52,417 ED visits were recorded in the study hospitals during a 6-month period. Table 2 presents a summary of the data showing the relative proportions of the 5 leading causes of ED visits. Motorcycle injuries were the second leading cause of ED visits, with a total of 1,073 accounting for 2.0% of the total emergency visits and 12.0% of all injuries that presented to the hospitals. They were also the second leading cause of injury admissions in hospitals (13.6%).

Motorcycle injuries were the second leading cause of ED visits.

**TABLE 2.** Types of Injuries at Hospital Emergency Departments in Kisumu, Kenya

	**ED Attendance, No. (%)**	**Hospital Admissions, No. (%)**
Assaults	2,368 (26.6)	813 (22.4)
Other road traffic accidents	658 (7.4)	398 (11.0)
Falls	987 (11.1)	433 (11.9)
Motorcycle injury cases	1,073 (12.0)	494 (13.6)
Other injuries^a^	3,815 (42.9)	1,416 (39.0)
Total	8,901	3,627

Abbreviation: ED, emergency department.

a Other injuries constitute burns, drownings, bites (e.g., dog, snake), poisonings, pricks, or attempted suicide.

### Sex and Age Characteristics

We stratified hospital usage by motorcycle crash injury cases by sex and 2 age-group categories, children aged 0–14 years and adults aged 15 years and older. We found significant overrepresentation of males in all categories of hospital attendees in all age groups (*P*<.001). The sex of motorcycle crash injury cases varied by age group, where a greater proportion of males was found in nearly every age group; however, the proportions of females were overrepresented for motorcycle injury crash cases admitted to the hospital (Table 3).

**TABLE 3.** Motorcycle Crash Injury Cases at Hospitals in Kisumu, Kenya, by Age and Sex

	**ED Visits, No.**		**Hospital Admissions, No.**		**Prehospital Deaths, No.**		**Total, No. (%)**	**OR (95% CI)**
	**Male**	**Female**	**Male**	**Female**	**Male**	**Female**		
Age, years								
<15	16	22	10	37	2	5	92 (8.6)	1.3 (1.1, 1.5)
>15	376	134	420	27	17	7	981 (91.4)	3.7 (3.1, 4.8)
Total	392	156	430	64	19	12	1,073 (100)	

Abbreviations: CI, confidence interval; ED, emergency department; OR, odds ratio.

### Hospital Services Usage by Motorcycle Crash Injury Cases

**FIGURE 1:**
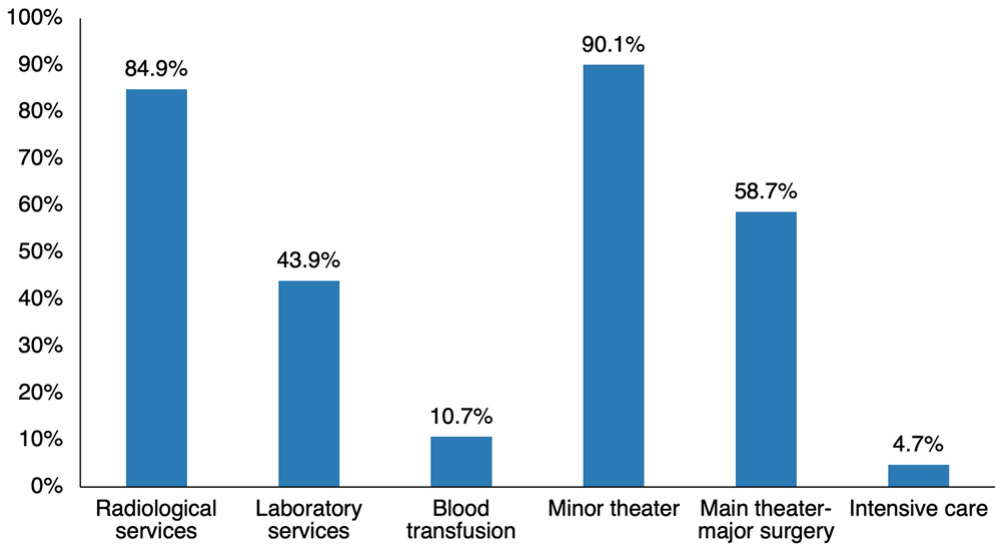
Utilization of Heath Care Services Motorcycle Injury Cases at Hospitals in Kisumu City, Kenya

Analysis of services used by the admitted motorcycle crash injury cases showed that 90.1% and 58.7%, respectively, required the operating theater for minor and major surgical procedures (Figure 1). Radiological services were used by 84.9% of the patients. In addition, of the 123 patients admitted to the ICU, 42.3% were due to motorcycle crash injuries.

### Injury Cases by Road User

Table 4 shows the distribution of types of road users among cases in the ED or admitted to the hospital. Pedestrians hit by motorcycles were the most numerous ED patients (208 [38.0%]), followed by motorcycle riders (168 [30.7%]). Among hospital admissions, motorcycle riders were most common (190 [38.5%]), followed by pedestrians (156 [31.5%]). Pillion passengers ranked third among both ED visits (29.8%) and admissions (29.0%).

**TABLE 4.** Motorcycle Crash Injury Cases at Hospitals in Kisumu, Kenya, by Type of Road User

**Type of Road User**	**ED Visits, No. (%)**	**Hospital Admissions, No. (%)**	**Total, No. (%)**	**95% CI**
Motorcycle rider	168 (30.7)	190 (38.5)	358 (34.4)	30.6, 39.0
Motorcycle passenger	163 (29.7)	143 (29.0)	306 (29.4)	28.7, 30.7
Pedestrian	208 (38.0)	156 (31.5)	364 (34.9)	29.3, 38.0
Bicyclist	9 (1.6)	5 (1.0)	14 (1.3)	3.3, 5.1
Total	548 (70.4)	494 (26.7)	1,042	

Abbreviations: CI, confidence interval; ED, emergency department.

### Injury Cases by Anatomic Site

The frequency distribution of the types of injuries, based on anatomic site of injury, is presented in Table 5. Injuries to the head and neck region (490 [47.0%]) comprised the majority of injury presentations, with more than half (285 [55.2%]) resulting in hospital admission. This was followed by injuries to the lower extremities (166 [20.5%]). Injuries to the pelvis and spine were uncommon, representing 8 (0.8%) and 2 (0.2%), respectively, of all ED visits and hospital admissions.

**TABLE 5.** Motorcycle Crash Injury Cases at Hospitals in Kisumu, Kenya, by Anatomic Site of Injury

**Injury Site**	**OPD Attendances, No. (%)**	**Hospital Admissions, No. (%)**	**Total, No. (%)**	**OR (95% CI)**
Head and neck	205 (37.7)	285 (57.7)	490 (47.0)	2.60 (0.35, 3.35)
Chest	26 (4.6)	22 (4.5)	48 (4.6)	0.04 (0.35, 0.80)
Abdomen	72 (13.1)	61 (12.9)	133 (12.7)	0.30 (0.46, 0.68)
Pelvis	8 (1.1)	0 (0)	8 (0.8)	0.90 (−0.40, 3.94)
Spine	1 (0.0)	1 (0.4)	2 (0.2)	1.20 (−4.26, 2.03)
Upper extremity	47 (8.6)	38 (7.7)	85 (8.1)	0.30 (0.55, 0.71)
Lower extremity	133 (24.6)	33 (6.6)	166 (20.5)	0.27 (0.09, 1.16)
Multiple sites	56 (10.3)	54 (10.9)	115 (10.6)	1.30 (0.91, 3.90)
Total	548 (52.6)	494 (47.9)	1,042	

Abbreviations: CI, confidence interval; OPD, outpatient department; OR, odds ratio.

### Distribution of Proportion of Motorcycle Crash Injury Admissions by Helmet Use

Analysis of the relationship between helmet use and hospital presentation by motorcycle riders and pillion passengers in Table 6 indicates that most motorcycle injury patients (73.6%) were not wearing a helmet at the time of the crash. Lack of helmet use was significantly associated with increased hospital admissions (odds ratio [OR]=8.2; 95% confidence interval [CI]=4.273, 17.539).

**TABLE 6.** Crash Injury Cases of Motorcycle Riders and Their Passengers at Hospitals in Kisumu, Kenya, by Helmet Use

**Helmet Use**	**ED Attendances, No. (%)**	**Hospital Admissions, No. (%)**	**Total, No. (%)**	**OR (95% CI)**
Yes	121 (33.7)	59 (18.3)	180 (26.4)	1.31 (1.01, 1.62)
No	238 (66.3)	264 (81.7)	502 (73.6)	8.2 (4.273, 17.12)
Total	359 (100)	323 (100)	682 (100)	

Abbreviations: CI, confidence interval; ED, emergency department; OR, odds ratio.

### Burden of Motorcycle Crash Injuries on Radiological Services in the Hospitals

**FIGURE 2:**
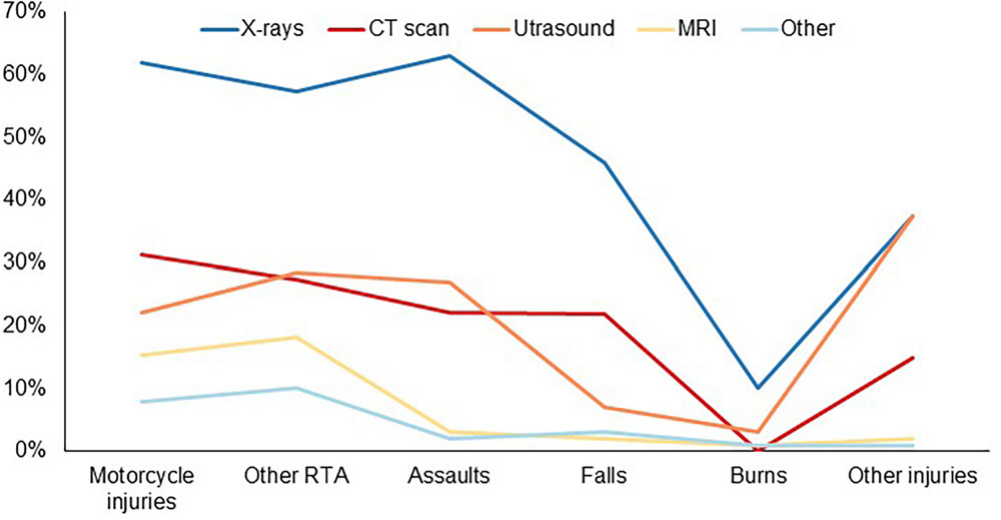
Burden of Motorcycle Injuries on Radiological Services in Relation to Other Injuries at Hospitals in Kisumu City, Kenya Abbreviations: CT scan, computerized tomography scan; MRI, magnetic resonance imaging; RTA, road traffic accident.

Of the total number of radiological investigations (3,502), 63.6% were X-rays. Motorcycle injury patients comprised 62% of all the patients receiving X-rays, and motorcycle injuries were the second leading cause of radiological investigations after assaults. Motorcycle injuries were the leading cause of computerized tomography (CT) scans, representing 31.3% (Figure 2). Usage of radiological services varied significantly with hospital attended (*P*<.001), severity of injuries (*P*<.001), type of road user (*P*=.002), medical/surgical procedures used (*P*< .001), alcohol use (*P*<.001), age of the injured patients (*P*<.001), and helmet use (*P*< .001).

### Distribution of Types of Radiological Services Among Motorcycle Crash Injury Patients

X-rays accounted for 82.1% of radiological services used and included imaging of patients with suspected limb fractures, chest injuries, head and maxillofacial injuries, and pelvic and spinal injuries. Other radiological services used included CT scan (82 patients), ultrasound (47 patients), and magnetic resonance imaging (10 patients) (Table 7).

**TABLE 7.** Distribution of Types of Radiological Services Among Motorcycle Crash Injury Patients at Hospitals in Kisumu, Kenya

**Service Type**	**Patients, No.** **(n=887)**
X-rays	748
CT scan	82
Ultrasound	47
MRI	10

Abbreviations: CT scan, computerized tomography scan; MRI, magnetic resonance imaging.

### Burden of Motorcycle Crash Injuries on Surgical Department

**FIGURE 3:**
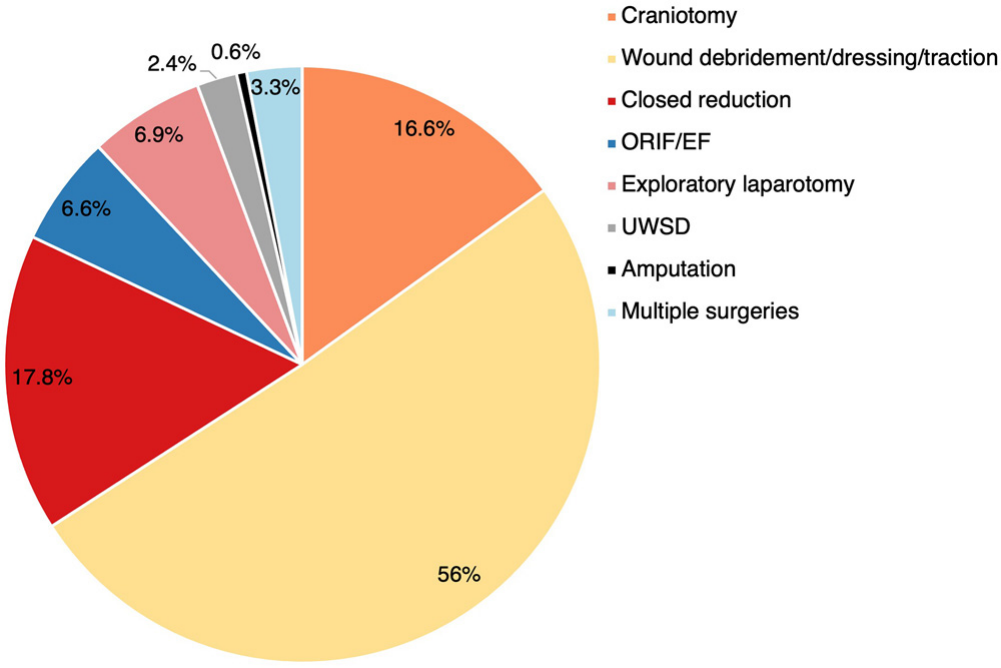
Isolated Medical/Surgical Procedures and Severity of Motorcycle Injuries at Hospitals in Kisumu City, Kenya Abbreviations: ORIF/EF, open reduction and internal fixation/external fixation; UWSD, underwater seal drain.

We conducted an analysis of motorcycle injury data on various surgical interventions undertaken by external cause of injury. Of the 3,652 surgical procedures conducted within the hospitals during the study period, 1,939 (53.09%) procedures were performed on the 939 motorcycle injury cases in the ED and admitted to the hospital. Altogether, 87.5% of motorcycle-related injuries required surgical interventions. Figure 3 shows the distribution of the various surgical procedures; wound debridement, suturing, and cleaning were the most common (51.0%). Six motorcycle crash injury cases were amputated at the lower extremities (Figure 3).

Overall, 87.5% of motorcycle-related injuries required surgical interventions.

### Injury Severity and Surgical Intervention

An assessment of the relationship between ISS and type of surgical intervention showed that patients with ISS levels ≥16 were more likely to undergo a craniotomy (OR=2.5; 95% CI=1.8, 4.3) or closed fracture reduction (OR=1.8; 95% CI=1.1, 2.5) than those with low ISS levels (<16) (Table 8).

**TABLE 8.** Relationship Between Surgical Procedures and Severity of Motorcycle Crash Injuries at Hospitals in Kisumu, Kenya

**Surgical Procedure**	**Patients With ISS <16, No. (%)**	**Patients With ISS ≥16, No. (%)**	**Total, No. (%)**	**OR (95% CI)**
Craniotomy	89 (13.0)	67 (25.9)	156 (16.6)	2.5 (1.8, 4.3)
Wound debridement/dressing	383 (56.3)	53 (20.5)	436 (56.0)	1.1 (0.2, 2.3)
Closed fracture reduction	119 (17.5)	47 (18.1)	166 (17.8)	1.8 (1.1, 2.5)
ORIF/EF	28 (4.1)	28 (10.8)	56 (6.6)	1.4 (0.2, 2.91)
Exploratory laparotomy	43 (6.3)	22 (8.5)	65 (0.6)	0.9 (0.1, 1.6)
UWSD	13 (1.9)	10 (3.9)	23 (2.4)	1.1 (0.6, 2.1)
Amputation	0 (0.0)	6 (2.3)	6 (0.6)	2.8 (1.2, 4.1)
Multiple surgeries	5 (0.7)	26 (10.0)	31 (18.4)	3.2 (1.4, 5.6)
Total	680 (100)	259 (100)	939 (100)	

Abbreviations: CI, confidence interval; ISS, injury severity score; OR, odds ratio; ORIF/EF, open reduction and internal fixation/external fixation; UWSD, underwater seal drain.

### Injury Severity and Admission Status

Analysis of patient disposition by mean ISS demonstrated significant overall differences between motorcycle injury patients treated in the ED and those who were admitted to the hospital. The mean ISS for motorcycle injury patients who were admitted was significantly higher (ISS≥16) than for those treated in the ED (ISS<16; *P*<.001). Mean ISS also differed significantly across all road users (*P*<.001); scores were greater for motorcycle riders and pedestrians (mean ISS=24.8 and 24.1, respectively) than for bicyclists and passengers (mean ISS=12.7 and 13.3, respectively).

### Injury Outcome and Transportation and Time Taken to the Hospital

Of the 1,073 motorcycle injury patients, only 73 (6.8%) were transported to the health facilities by ambulance; the majority (702 [65.4%]) arrived by taxi or motorcycle. Transportation by a taxi or motorcycle was significantly associated with higher odds for severe and fatal injuries compared to those who arrived by ambulances ([OR=1.630; 95% CI=0.915, 2.34; *P*=.0021] vs. [OR=0.462; 95% CI=0.381, 0.933]). Of all the patients with motorcycle crash injuries, only 318 (29.6%) arrived at the health facilities within 1 hour from the time of the accident. The mean travel interval from time of accident to arrival at the hospital was 3.92 hours. Arrival at the hospital after 1 hour was associated with higher odds for severe head injuries (OR=1.9; 95% CI=1.626, 3.981) and femoral bone fractures (OR=1.662; 95% CI=0.846, 2.960).

### Medical Cost

The total medical cost for motorcycle crash injury patients was 19,134,871 Kenyan shillings (Ksh) (US$1,913,487.71). The cost of medication was Ksh200–Ksh1,035,698 (US$1–US$10,356.98). Of the total medical cost, 48.7% was spent on surgical interventions. The medical cost was also dependent on the type of intervention or procedure performed. Costs were particularly higher for craniotomies (40.8%), open reductions (25.1%), and laparotomies (11.5%) and for those admitted to the ICU for extended periods of time. Radiological investigations, including X-rays, CT scans, and magnetic resonance imaging, accounted for 18.5% of the total medical cost (Table 9).

**TABLE 9.** Medical Cost^a^ of Services Offered to Motorcycle Crash Injury Patients at Hospitals in Kisumu, Kenya

	**Median** **Cost, Kenyan Shillings**	**Minimum Cost per Case, Kenyan Shillings**	**Maximum Cost per Case, Kenyan Shillings**	**Total** **Cost, Kenyan Shillings**	**% (95% CI)**
Cost of services offered to motorcycle crash injury patients					
Laboratory investigation	589	1200	8040	423730	2.2 (1.8, 2.6)
Radiological investigation	3965	400	61040	3505000	18.3 (17.8, 19.9)
Minor surgery	1210	400	2500	902374	4.7 (4.1, 5.3)
Major surgery	27773	5000	525626	8428708	44.0 (41, 48)
Medication and physician services	1061	200	65734	2861329	15.0 (13.2, 16.8)
Admission and ICU	765	1200	355657	2101727	11.0 (9.0, 13.0)
Others	241	200	7000	912019	4.8 (2.6, 6.9)
Total		200	1,033700	19134887	
Cost of surgical procedures offered to motorcycle crash injury patients					
Debridement/cleaning and dressing	1198	400	78750	593055	7.0 (3.4, 10.6)
Craniotomy	29904	5000	300000	3438913	40.8 (38.9, −42.9)
Closed reduction	2704	600	130000	402820	4.8 (2.5, 7.3)
Exploratory laparotomy	17879	7000	130000	965468	11.5 (9.6, 12.4)
ORIF/EF	29851	4000	117000	2119435	25.1 (22.6, 27.6)
UWSD	47172	3000	30000	849089	10.1 (8.1, 22.1)
Others	9988	15237	25667	962301	11.4 (10.2, 12.8)
Total				8428708	

Abbreviations: CI, confidence interval; ICU, intensive care unit; ORIF/EF, open reduction and internal fixation/external fixation; UWSD, underwater seal drain.

a 123 Kenyan shillings=US$1.

## DISCUSSION

A review of the previous studies from Kenya and other LMICs revealed the dearth of data on the burden of motorcycle injuries on the health care system. Our study is the first to establish the burden of motorcycle injuries on the public health system in both Kenya and the World Health Organization Africa region. The high proportion of motorcycle crash injuries due to increased use of motorcycles for public transport is attributed to behavioral practices. These injuries exert an additional burden on Kenyan public health services.

Assessment of hospital service usage among the admitted cases showed that motorcycle injury cases require critical clinical services, including close monitoring by clinicians, radiological investigations, and major surgical procedures. Compared to assaults and other injuries, motorcycle injuries required a higher proportion of radiological services, operating theater services for minor and major surgeries, laboratory investigations, transfusions, and ICU services, making them a leading cause of demand for hospital services. These injuries impose a resulting strain on the Kenyan health care system because of the need for expensive surgical and ICU facilities, skilled professionals, advanced technology, and substantial amounts of medical commodities. Traumatic injuries such as these can also be disruptive because regular services may need to be postponed to attend to critically injured victims.^42^

Assessment of hospital service usage showed that motorcycle injury cases require critical services including radiological investigations and major surgical procedures.

Motorcycle injury to the head and neck region (42.5%) comprised the bulk of injury presentations, with more than half (55.2%) resulting in hospitalization. Head injuries impose a substantial burden on the health care system. This finding should be considered in the planning of emergency trauma service delivery, especially in reference to human resources, bed capacity, equipment, and supplies.

Not wearing a helmet impacted the likelihood of hospital admission for motorcycle injury patients. Compared to patients who had worn helmets during the crash, a greater number of motorcycle injury patients who did not wear helmets presented with high Glasgow Coma Score (≤7) and ISS (≥16) with increased odds of hospital and ICU admission.

Prevention of head injuries and lower limb injuries would require advocacy on measures such as wearing protective helmets, protective boots, kneepads, and padded gear. Evidence demonstrates the effectiveness of enactment and enforcement of helmet laws in increasing helmet use and reducing head injuries. However, legislation without effective enforcement is not adequate.^1^ For example, in Kenya, the Traffic Amendment Bill passed by legislators in 2009 and the Traffic Amendment Act in 2012 constituted mandatory helmet use for all motorcycle riders and their passengers.^25^^,^^26^ However, studies from 2017 and 2018 on helmet use in Kenya reported average helmet use prevalence of 36.3% and 28%, respectively, which indicates very little improvement following legislative implementation.^23^^,^^24^^,^^27^ Bachani et al. report that motorcycle users do not wear helmets for various reasons, including considering it unhygienic to share, inconvenient, or uncomfortable.^27^ Understanding all the fundamental reasons for low helmet use will be a prerequisite to the development of targeted approaches to improve uptake.

In addition to implementing safety interventions, the World Health Organization has recommended the implementation of emergency medical systems to improve injury care and outcomes in LMICs.^1^ Studies have shown that early quality prehospital care and hospital care prevent and reduce the probability of death and adverse sequelae.^30^^,^^31^ However, the prehospital care system that facilitates emergency transportation and injury management in the prehospital setting is inadequate in Kenya. This partly could explain why there were high proportions of prehospital deaths in this study and only 6.8% of the motorcycle injury patients arrived at the hospital by ambulance. Hospital arrival after 1 hour was associated with a higher likelihood of severe head injuries and femoral bone fractures and of high mortality.

ISS provides a valid measure of morbidity status of a casualty and the prognosis of survival or death from multiple injuries.^29^ Patients with low ISS have fewer types of injuries in general and recover more rapidly, whereas those with higher scores are more likely to have a longer period of recuperation. Previous studies by Ali and Shepherd and Gardner et al. provide consistent evidence of the association between injury severity and hospitalization regarding motorcycle injury patients.^43^^,^^44^ This implies that hospitalization may be used as a proxy measure of injury severity. However, this needs to be cautiously applied for 3 reasons: (1) some patients with minor injuries may remain in the hospital for more days due to other complications; (2) other patients may be detained for personal reasons (such as inability to promptly settle hospital bills or not having bus fare or relatives nearby to help with transportation); and (3) seriously injured patients may stay for only a short period before being transferred to tertiary hospitals, such as those with ICU facilities for specialized care. It is also important to note the potential limitations when comparing hospitalization rates between different types of hospitals. For example, a private hospital that exists to make a profit may have a tendency to retain patients with less severe injuries for disproportionately longer days, which is likely to falsify the relationship between ISS and the actual hospitalization. Therefore, it would be useful to establish and maintain minimum admission criteria, standardized inpatient management practices, and patient discharge procedures across all hospitals, both private and public, to improve the validity of using hospitalization as an indicator of both injury severity and hospital usage. Hospitalization and discharge destination are significant outcome measures used in public health system evaluation.^45^^,^^46^

The mean ISS between in- and outpatients also differed significantly across all types of road users and was greater for motorcycle riders and pedestrians than for bicyclists and passengers. This pattern gives some indication of the relative importance of each injury mechanism in terms of the likelihood of hospitalization, survival, or death, as well as the range and amount of resources likely to be required for treatment. And for those affected by traffic trauma, a greater proportion of motorcycle riders and pedestrians sustain more severe forms of injuries than other road users; motorcycle-pedestrian and motorcycle–motor vehicle collisions are more likely to be fatal or critical (due to accident impact force).

A striking feature of this study is the demonstration of the extent to which motorcycle crashes contribute to increased usage of public health systems resources. Motorcycle injuries are the single most important cause for radiological diagnostic services such as X-rays, CT scans, magnetic resonance imaging, and ultrasound. Similar findings have been reported in other studies in Tanzania and Nigeria.^19^^,^^47^^,^^48^ Due to the dynamics of motorcycle trauma and the numbers involved, the urgency and priority for X-ray demand result in immediate overload in the department at the expense of patients with other conditions.

Our results showed that motorcycle injuries are the single most important cause for radiological diagnostic services.

Motorcycle injuries are characterized by the high usage rate of operating theaters (approximately 53.0% of all major surgeries), which has implications for the costs of medical services in terms of resource inputs and staff requirements. The study revealed that motorcycle injuries accounted for 96.1% of major surgeries among hospitalized cases and 80.3% of minor surgeries among outpatients. The numbers of motorcycle injury cases demanding minor or major surgical procedures are substantial (87.5%), leading to overcrowding in the ED and surgical wards. This finding is similar to those reported in Brazil by Liu et al. and Calgary, Canada, by Rifaat et al., that showed motorcycle injuries had high proportions demanding surgical intervention.^9^^,^^49^

Studies suggest that the financial burden associated with motorcycle injuries in high-resource settings is significant.^50^ Data on the cost of motorcycle injuries in Africa is minimal. This study endeavored to estimate the burden of motorcycle injuries on the health system, including the medical cost incurred. Motorcycle injuries use 3.7% and 3.2% of the 2 public hospitals’ annual budgets, which represent large sums of money to spend on a single preventable health problem. The medical costs of motorcycle crash injuries contribute to high direct costs, which has been shown to take up the greatest portion of total costs in cases where the victim survives.^51^^,^^52^ However, motorcycle injuries also incur indirect costs in terms of lost productivity that outweigh direct costs due to consequent disabilities and treatment, emphasizing the long-term effects of these injuries on the economic welfare of society.^51^ The resulting reduced productivity and long-term disability suffered by the victims make them a social and financial burden to families and society at large.^52^ The medical costs from motorcycle injuries are incurred by different entities, including the victims, private insurers, and the government. Therefore, the insured members of the public, taxpayers, insurers, and governments should have an interest in quantifying not only the amount of these motorcycle injury costs but also their impact on the economy and public health system.

Motorcycle injuries incur indirect costs in terms of lost productivity that outweigh direct costs due to consequent disabilities and treatment, emphasizing the long-term effects of these injuries on the economic welfare of society.

### Strengths and Limitations

The major strength of this study is that all motorcycle crash injuries that presented to Tier III hospitals in Kisumu City were enrolled and generated comprehensive data on the burden of motorcycle crashes on health services. Capturing data on actual motorcycle injury cases also ensured the accuracy of the information and avoidance of recall bias. The use of a priori analysis allowed for the statistical power and the study effect size to be determined before the actual study was conducted. An a priori analysis provides an efficient method of controlling statistical power before a study is actually conducted.^32^

These results should be considered in light of a study limitation. This study focused only on motorcycle injury patients who sought and obtained care in 3 major referral hospitals, and nothing is known about those seeking care elsewhere. Therefore, motorcycle injury cases are likely to be underestimated. Nevertheless, useful data on injured patients choosing to seek care at the hospitals could be obtained and the consequent burden on the hospitals quantified. However, these hospitals were chosen deliberately to focus on motorcycle casualties with injuries severe enough to demand hospital services in EDs because these departments provide the best opportunity for capturing data on a wide range of nonfatal injuries.

## CONCLUSION

Although hardly recorded in routine hospital statistics, motorcycle-related injuries are a major strain on the health care systems in many LMICs. The findings of our study illustrate the magnitude of hospital burden due to motorcycle injuries and the associated factors. The use of helmets among motorcycle riders and passengers was low and was significantly associated with hospital admissions. Motorcycle injuries are preventable and their consequent burden on health systems can be curtailed. There is an urgent need to identify, promote, and effectively implement suitable interventions focused on motorcycle safety.

## Notes

**Peer Reviewed**

**First published online:** January 11, 2023.
